# The Effects of Synthetic Polymers on the Release Patterns of Bupivacaine Hydrochloride from Sodium Hyaluronate Hydrogels

**DOI:** 10.3390/biomedicines13010039

**Published:** 2024-12-27

**Authors:** Dorota Wójcik-Pastuszka, Roksana Iwaszkiewicz, Witold Musiał

**Affiliations:** Department of Physical Chemistry and Biophysics, Faculty of Pharmacy, Wroclaw Medical University, ul. Borowska 211A, 55-556 Wrocław, Poland; dorota.wojcik-pastuszka@umw.edu.pl (D.W.-P.); roksanaiwaszkiewicz@gmail.com (R.I.)

**Keywords:** bupivacaine hydrochloride, sodium hyaluronate, intra-articular hydrogel, viscosity study, release kinetics

## Abstract

Background: Using hydrogels for the controlled release of drugs is beneficial for patients, who then receive the proper dose of the medicinal substance. In addition, the formulation can provide more consistent drug absorption while reducing the frequency of dosing. Objectives: The aim of this investigation is to propose a novel HA (sodium hyaluronate)-based hydrogel for intra-articular injection doped with synthetic polymers and incorporated with bupivacaine hydrochloride (Bu) as a local anesthetic. The other aim of this study is to reveal the effects of the formulation’s ingredients on its viscosity and the relationship between the hydrogel’s viscosity and drug release. Methods: First, HA-based hydrogels doped with synthetic polymers and incorporated with Bu were prepared. A study of the hydrogels’ viscosities was performed using a rotational viscometer. Release tests were carried out by employing a paddle-over-disk apparatus following the USP/Ph.Eur guidelines. The drug concentrations in the acceptor fluid were analyzed spectrophotometrically. Results: It was found that the viscosity of the hydrogels doped with synthetic polymers was higher than the viscosity of the hydrogels made with only HA. The viscosity of the hydrogels doped with AX (ammonium acryloyldimethyltaurate/VP copolymer) was the highest, measuring 6750 ± 160 cP and 12623 ± 379 cP with and without Bu, respectively. The results of the kinetic experiment indicate that the Higuchi and Korsmeyer–Peppas models best described the drug release. Bu was released the most slowly from the formulation doped with AX. The release rate constants obtained from the Higuchi and Korsmeyer–Peppas models were k_H_ = 4.4 ± 0.2 mg × min^−1/2^ and k_K-P_ = 3.4 ± 0.2 × 10^−2^ min^–N^, respectively. The half-release time, calculated using the Higuchi model, was the longest for the formulation doped with AX, at 199.5 ± 17.6 min. Conclusions: This indicates that the incorporation of AX into the hydrogel may prolong the drug dissolution. The hydrogel doped with AX was the optimal formulation for the controlled release of Bu.

## 1. Introduction

**Figure 1:**
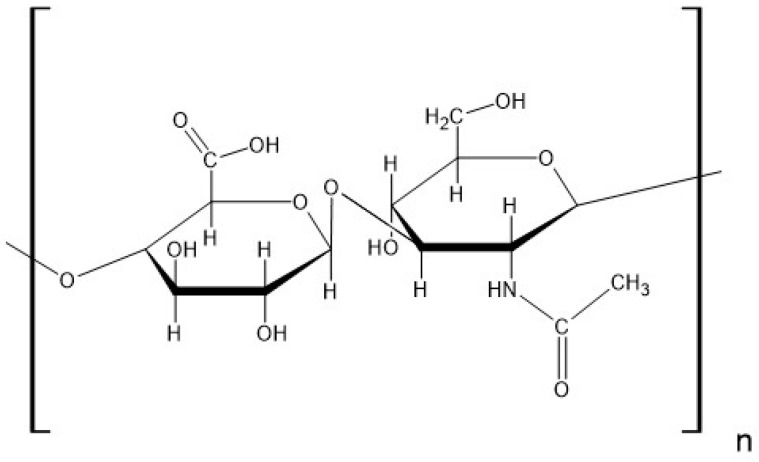
The structure of HA.

Hydrogels are composed of hydrophilic, crosslinking polymers that may form a three-dimensional network. This network has the ability to collect large amounts of water or biological fluids and allows other molecules, e.g., drugs, nutrients, and supplements, to be incorporated inside of the polymeric matrix. This structure may be employed for the delivery of various substances to a specific site of action [1,2]. Additionally, this system allows for the controlled release of the implemented substances. This often results in a reduced drug dosing frequency, stable blood levels of the substance, and a reduction in side effects [3]. Natural polymers, such as sodium hyaluronate (HA), provide hydrogels with low toxicity and high biocompatibility and biodegradation. HA is a polysaccharide composed of units of D-glucuronic acid and N-acetyl-D-glucosamine. The structure of HA is presented in Figure 1 [4].

The occurrence of polar and non-polar fragments in the structure of HA provide the possibility of chemical interactions between HA units and different chemical substances. Additionally, the formation of hydrogen bonds between HA units ensures the formation of secondary and tertiary structures [4,5]. It was found that the formation of an HA network depends on the molecular weight of the polymer and its concentration. The increase in the HA concentration and its molecular mass promotes the development of a three-dimensional network. However, in highly dilute solutions, the biomolecules occur individually [6]. The intramolecular polymer network can absorb large amounts of water, resulting in the high viscosity of the hydrogel. It was found that higher molecular weights of HA solutions corresponded to higher viscosity. Moreover, the degree of crosslinking in the polymer can also affect the rheological behavior of HA [7]. The viscoelastic properties of hydrogels enable their widespread usage, e.g., in medicine, pharmacy, and cosmetology, in wound treatment preparations, dermal filler, and eye drops, and for intra-articular injections.

Intra-articular injections are employed for the treatment of degenerative joint disease (OA, osteoarthritis). This method of therapy is called a viscosupplementation. Hyaluronan hydrogels have become popular for intra-articular injections because OA reduces the amount of this biomolecule in the synovial fluid. HA injections supplement the resulting deficiencies, improving cushioning and joint mobility and reducing inflammation and pain. Viscosupplementation is worth considering when oral treatments prove insufficient. Intra-articular injections can deliver substances directly to the joint, avoiding side effects. Other advantages of intra-articular delivery include increases in the local bioavailability, a reduction in systemic exposure, and fewer adverse events. Viscosupplementation is a good alternative for preventing the progression of changes already present in the joints [8,9]. Viscosupplementation can effectively postpone the need for surgery. Abate et al. [10] used 2% HA hydrogels for intra-articular injections in the treatment of knee osteoarthritis over a period of 4 months. However, a longer duration of HA administration resulted in a decrease in the therapy’s effectiveness.

**Figure 2:**
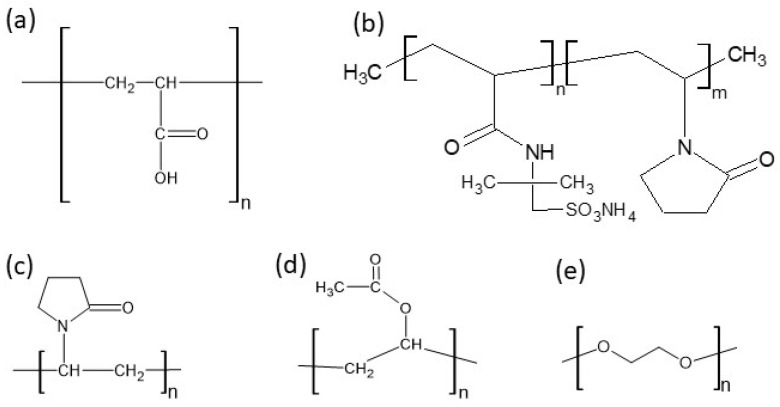
The structures of (**a**) polyacrylic acid—PA, (**b**) ammonium acryloyldimethyltaurate/VP copolymer—AX, (**c**) polyvinylpyrrolidone—PVP, (**d**) polyvinyl acetate—PVA, and (**e**) polyethylene glycol—PEG.

The properties of hydrogels may be modified by the incorporation of a second gelling agent that may be beneficial for the properties of the final products. Copolymer-based systems consisting of two or more different monomers may have additional values in comparison to the preparations composed of one polymer. The modification of the physical properties results from the composition of the hybrid products and the interactions among the ingredients [11]. Copolymers may form interpenetrating network (IPN) hydrogels that possess improved biocompatibility, mechanical strength, stability, and permeability. In drug delivery systems, IPNs can ensure controlled and sustained drug release due to their structural patterns and physicochemical properties [1]. Compositions based on amidated pectin (APN) doped with synthetic polymers, such as polyacrylic acid (PA), ammonium acryloyldimethyltaurate/VP copolymer (AX), polyvinylpyrrolidone (PVP), and polyethylene glycol (PEG), have been proposed as mesalazine carriers for oral administration. The structures of these synthetic polymers are presented in Figure 2 [12,13,14,15,16].

In previous studies, we revealed the interaction between APN and synthetic polymers, which influenced the drug release rate from the preparations [17,18]. Wei et al. [19] proposed a new injectable hydrogel for wound therapy based on modified gelatin and modified HA doped with polydopamine (PDA) nanoparticles. The evaluated hydrogel had good antibacterial and antioxidant properties, and the used biopolymers provided very high biological compatibility.

One example of a copolymer-based hydrogel is poly(acrylic acid)-co-poly(ethylene glycol) (PA-co-PEG), which is composed of acrylic acid and ethylene glycol. The incorporation of acrylic acid to this system provides it with pH-sensitive swelling properties. The PA-co-PEG hydrogel was applied in drug delivery systems where pH-dependent dissolution profiles were required [1]. Another example of a hydrogel consisting of two polymers is HA blended with modified polyvinylpyrrolidone (PVP). A study determined the interaction between these macromolecules, and the combination of these polymers resulted in an elastic composition with better properties than the homopolymer composition [20]. Zhang et al. [16] studied an injectable hydrogel composed of methacrylated HA (HA-ene) and 4-arm PEG-SH, which was used as a crosslinking agent. The addition of 4-arm PEG-SH improved the rheological properties of the preparation due to the bond formation between the sulfhydryl groups of 4-arm PEG-SH and the methacrylic groups of HA-ene. The obtained hydrogel sustainably released small doses of verteporfin into wound microenvironments. In addition to changing the rheological properties of the hydrogel, PEG also ensured biocompatibility, low toxicity, and high solubility in water [21].

**Figure 3:**
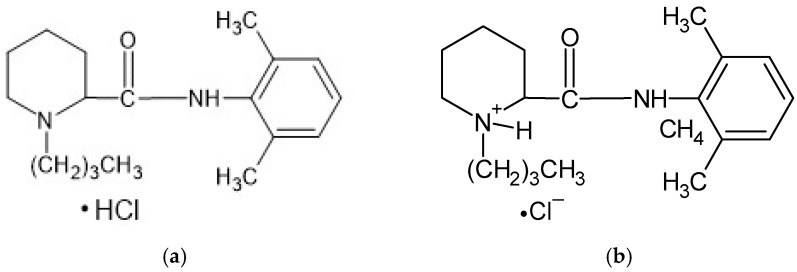
The structure of bupivacaine hydrochloride (**a**) in the free-base form, and (**b**) in the ionized form.

According to He et al. [22], HA present in a joint cavity has a short half-life, limiting its use as a biocompatible, biodegradable, and non-immunogenic compound. However, chemical modification of the carboxyl and hydroxyl groups of glucuronic acid and crosslinking of the biopolymer may prolong the retention time of HA, making it an attractive carrier for intra-articular injections. This drug application method is widely used in corticosteroids, steroidal anti-inflammatory drugs, and non-steroidal anti-inflammatory drugs (NSAIDs). Local anesthetics (LAs) such as lidocaine, bupivacaine, and ropivacaine are commonly used in joint therapy; however, they are usually administered in the form of a suspension [23,24]. These preparations clear rapidly from the joint space after injection, often within 30 min [25]. LAs are a group of drugs whose mechanism of action is to block voltage-gated sodium channels. They are composed of a lipophilic aromatic group connected by an alkyl chain to an amide group. Either ester bonds or amide bonds occur in LA molecules. Ester bonds undergo hydrolysis, making such drugs unstable, while amide bonds are more stable, which leads to a longer duration of action and slower biotransformation of the drug. The structure of Bu is presented in Figure 3 [26,27].

Santamaria et al. [28] reported that HA can bind to LAs, and their obtained formulation had a longer duration due to the increased viscosity of the polymeric hydrogel. Moser et al. [29] investigated the cytotoxicity of LAs and found that the combination of LAs with HA and glucocorticoids (GCs) reduced their cytotoxicity compared to LA alone and LA/GC.

We have previously studied the viscosity of hydrogels based solely on HA and the release of drugs from these formulations [30,31]. It is important to determine the effects of an additional polymer on both the viscosity of hydrophilic gels and the drug release from the preparations in order to propose an optimal composition for the prolonged release of Bu. Studies on the release of mesalazine from a composition based on pectin with the addition of a second polymer, PA, showed that this was the optimal formulation for the prolonged release of mesalazine [17,18]. Therefore, it will valuable to determine which of the applied doped polymers can provide the prolonged release of Bu from HA-based hydrogels.

The aim of this work is to propose a novel intra-articular hydrogel based on HA doped with synthetic polymers, such as PA, AX, a PVA-PVP mixture, and PEG incorporated with bupivacaine hydrochloride as a local anesthetic. Another purpose of this investigation is to reveal the effects of incorporating Bu into the carrier on its viscosity, the influence of the additional polymer on the hydrogel’s viscosity and the drug release, and the relationship between the formulation’s viscosity and the drug dissolution from the obtained preparations.

## 2. Materials and Methods

Bupivacaine hydrochloride (Bu) was purchased from POL-AURA (batch 189OTZ, Morąg, Poland). High-molecular-weight sodium hyaluronate (HA) with a molecular weight of >1.10 MDa was bought from ESENT (series no. 20230912, Szczecin, Poland). Polyacrylic acid (Carbopol^®^ 980NF, PA) was obtained from Lubrizol (batch number 0102457428, Wickliffe, OH, USA). Polyethylene glycol 4000 (PEG) was purchased from POL-AURA (batch number CHJ594, Morąg, Poland). Ammonium acryloyldimethyltaurate/VP copolymer (Aristoflex AVC, AX) was purchased from Clariant International Ltd. (batch number 13824026892, Muttenz, Switzerland). A polyvinyl acetate–polyvinylpyrrolidone mixture (PVA-PVP, kollidon^®^ SR) was obtained from Merck (batch number 16090424U0, Darmstadt, Germany). Trisodium phosphate dodecahydrate was obtained from CHEMPUR (batch number 16/03/01, Piekary Śląskie, Poland). Hydrochloric acid 35–38% was bought from Avantor Performance Materials (batch number 1023/12/13, Piekary Śląskie, Poland). Semi-permeable cellulose filters were bought from Carl Roth (Karlsruhe, Germany).

### 2.1. Hydrogel Preparation

**Figure 4:**
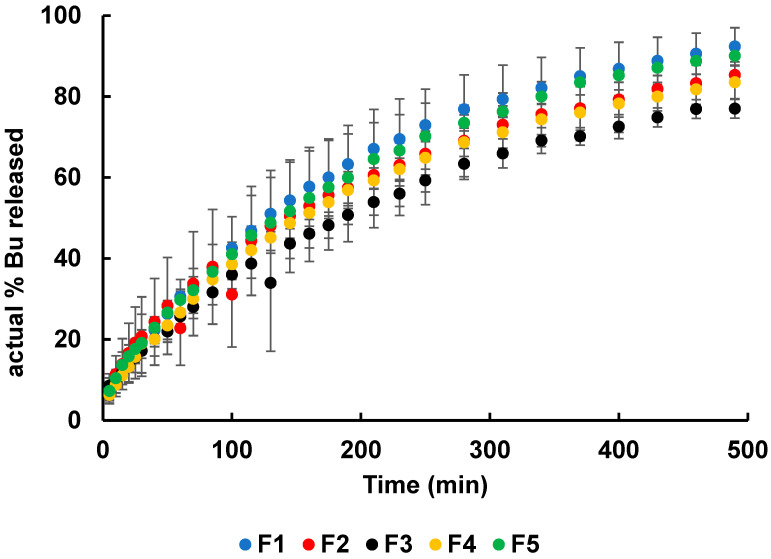
The dissolution curves of Bu in formulations F1–F5; n = 6.

Bu was dissolved in water according to the compositions shown in Table 1. The required amount of HA and a synthetic polymer (as presented in Table 1) were placed in the mortar and mixed manually with the Bu solution. The obtained mixture was homogenized using the Unidrive X 1000D homogenizer (Cat, Staufen, Germany), with a rotation speed of 16,000 rpm for 5 min. The compositions of the hydrogels are presented in Table 1, and pictures of the obtained preparations are shown in Figure 4.

The obtained preparations were stored at a temperature of 6 °C for 24 h to remove the air bubbles.

The Bu suspension for the injections of local anesthesia was prepared in concentrations of 0.25%, 0.5%, and 0.75%. The highest single dose of Bu for adults in local infiltration is 175 mg for preparations with a concentration of 0.25% [32]. This corresponds to 0.4375 mg of Bu, which is present in 29.1666 mg of the proposed hydrogels. The dose of Bu for an epidural block to achieve complete motor block in adults ranges from 75 to 150 mg for preparations with a concentration of 0.75% [32]. This means that 0.5625 mg to 1.125 mg of Bu is required, which corresponds to 37.5 mg to 75.0 mg of the hydrogels. The proposed formulations F1-F5 can deliver the amount of Bu commonly used in local anesthesia.

HA hydrogels used for intra-articular injections contain different concentrations of HA, e.g., 1% (20 mg/2 mL), 2% (40 mg/2 mL), and 1% (10 mg/1 mL) [33]. Temple-Wong [34] reported that the concentration of HA required in a human knee was 2.2 ± 1.6 mg/mL. Gupta et al. [35] revealed that the adult knee contains about 2 mL of synovial fluid with an HA concentration of 2.5–4.0 mg/mL.

Larrañeta et al. [36] proposed HA-based hydrogels obtained from aqueous mixtures containing 5% (*w*/*w*) HA and several different concentrations of Gantrez S97 (GAN): 0.5%, 1%, 3%, and 5% (*w*/*w*). In our previous work [17] on pectin milibeads doped with the same synthetic polymers used in the present study, we applied an additional polymer concentration of 1% (*w*/*v*).

All the ingredients, presented in Table 1, are commonly used in medicine. Bu is employed as an injectable suspension, mainly in surgery [37]. This drug is a local anesthetic used for caudal, epidural, and spinal anesthesia, and it is widely clinically applied in the treatment of acute and chronic pain. It can also block sodium channels and influences the activity of many other channels, including NMDA receptors [38]. Moreover, it has been shown to have antioxidant properties [39]. HA is used, among other applications, as a hydrogel for intra-articular injections to provide pain relief [40]. This biopolymer has demonstrated significant biological activity in skin care, orthopedic repair, and the monitoring of gynecological cancers [41]. PA is a popular drug delivery polymer, as it is non-toxic, biocompatible, and biodegradable [42]. AX, as a bioadhesive compound, was recently proposed in dentistry to help maintain the physical properties of enamel [43]. AX was also introduced into a vaginal formulation as a carrier of a biological bacteriostatic agent (BBA) [44]. PVA-PVP mixtures are widely applied in the pharmaceutical industry. They are used in formulations to prolong the drug release, improve the solubility and bioavailability, and stabilize the active ingredient [45]. Li et al. [46] carried out in vitro and in vivo studies of a PVP-based carrier with encapsulated apocynin and found that this system is a good carrier for ocular drug delivery. An injectable bioactive PEG-based hydrogel was recently proposed by Lao et al. [47] as a new type of a bone substitute with high potential for future clinical applications.

According to the FDA’s Voluntary Cosmetic Registration Report (VCRP) from 2018, PVP is the polymer most commonly used in cosmetic products. The second most commonly used polymer was reported to be ammonium acryloyldimethyltaurate/VP copolymer (AX). In a study involving rabbits, it was found that AX, when applied as a pure component in aqueous dispersion, was non-irritating to the skin of animals. The EFSA (European Food Safety Authority) Panel on Food Additives and Nutrient Sources added to Food has concluded the use of VP/VA copolymer in solid food supplements as a binding/coating agent does not pose a security risk. Another study reported that VP/VA copolymer was not a sensitizer in guinea pigs after repeated intracutaneous injections. AX was also not sensitizing in guinea pigs. The ocular irritation potential of VP/VA copolymer was also assessed, with ocular irritation observed at concentrations of 2.4% and 24% VP/VA copolymer, whereas minimal to moderate ocular irritation was observed at a concentration of 4%. The concentrations of 0.25%, 0.5%, and 1.75% were non-irritating. Additionally, it was found that AX was non-irritating to the eyes of rabbits. VP/VA copolymer was administered through the diet of rats. None of the animals died, and no clinical signs of toxicity were observed [48].

### 2.2. Viscosity Measurements

The obtained hydrogels were heated to 37 °C using the water bath (W215C, Labo Play, Bytom, Poland). At this temperature, viscosity measurements were performed using a rotational viscometer (DV2T, Brookfield, MD, USA). The viscosity study was carried out using spindle no. 4 at a rotational speed of 200 rpm for 1 min. All formulations were tested 6 times, and the mean dynamic viscosities with the standard deviations were obtained. The dynamic viscosity of each hydrogel at room temperature (25 °C) was measured using the same procedure as at 37 °C; however, spindle no. 3 was used because the viscosities were higher. The pH of each formulation was determined at room temperature (25 °C) using a pH meter (CX-601, ELMETRON, Zabrze, Poland) connected to the HYDROMET electrode (ERH-11S, Gliwice, Poland). Each preparation was measured 6 times, and the mean values of pH were calculated.

### 2.3. Dissolution Tests

The release of Bu was studied using a paddle apparatus, with six extraction cells placed at the bottom of the dissolution vessels [49]. The required amount of hydrogel was introduced into the donor chamber and covered with a cellulose membrane. Bu was mixed into 900 mL of phosphate buffer solution with a pH of 6.8, prepared according to Polish Pharmacopoeia XII [50]. The pH of the acceptor fluid reflected the pH of inflamed synovial fluid [51]. The measurements were carried out at a temperature of 37 °C and a paddle rotation speed of 50 rpm using the dedicated apparatus (ERWEKA, DT 126 Light, Heusenstamm, Germany). Then, 3 mL samples of the acceptor fluid containing the dissolved drug were collected at defined time intervals, followed by replenishment with the same volume of fresh medium. The absorbance of Bu was recorded using a UV-Vis spectrophotometer (Jasco V-530, Tokyo, Japan) at 263 nm. At this wavelength, the employed polymers were invisible; their spectra are included in the Supplementary Materials: Figures S1–S5. The Bu concentration was calculated using the obtained calibration curve. The actual % of the drug released at each time point was calculated. Each formulation was tested six times.

### 2.4. Difference Factor and Similarity Factor

In accordance with FDA recommendations [52], the curves of the mean percentage of Bu released versus time were compared by determining the values of the difference factor f_1_ and the similarity coefficients f_2_. The profiles of Bu release from formulations F2–F5, doped with synthetic polymers, were compared with the profile of Bu dissolution from F1, which was a reference formulation and did not contain a second polymer. Additionally, the release data from F2–F5 were also compared to test the impact of the polymer type on the release behavior of the drug.

### 2.5. Kinetic Study

**Figure 5:**
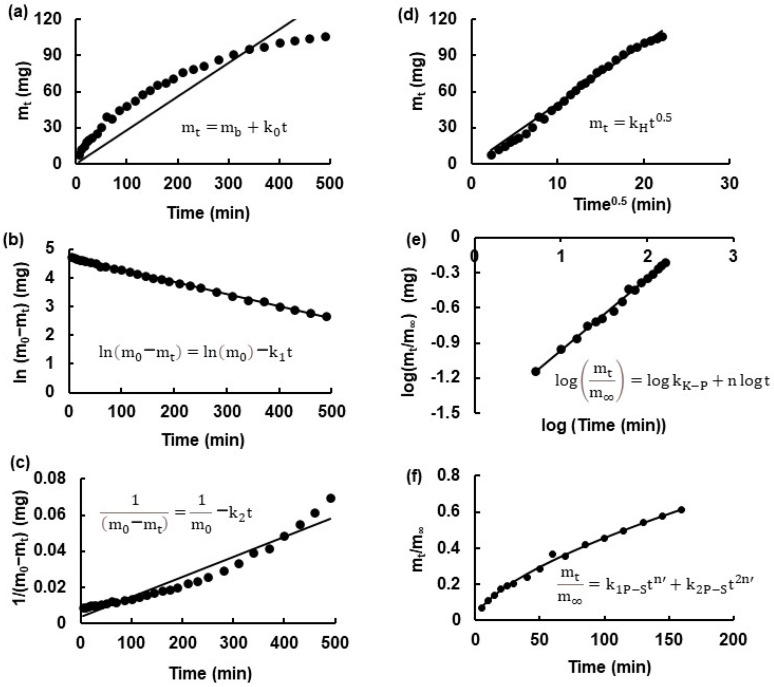
The theoretical curves fitted to the experimental points from the Bu release data from formulation F1 based on (**a**) zero-order kinetics, (**b**) first-order kinetics, (**c**) second-order kinetics, (**d**) the Higuchi model, (**e**) the Korsmeyer–Peppas equation, and (**f**) the Peppas–Sahlin model. m_t_—the amount of the drug released in time t; m_b_—the amount of the drug in the solution before it was released (usually 0); m_0_—the amount of the drug in the formulation before dissolution, m_∞_—the amount of the drug released after an infinite time t; k_0_, k_1_, k_2_, k_H_, and k_K-P_—the zero-, first-, and second-order, and Higuchi and Korsmeyer–Peppas release rate constants, respectively; n—the parameter indicating the drug release mechanism in the Korsmeyer–Peppas equation; k_1P-S_—the Peppas–Sahlin release rate constant for the Fickian contribution to drug release; k_2P-S_—the Peppas–Sahlin release rate constant for the Case II contribution to the drug release; n’—the diffusional exponent in the Peppas–Sahlin equation.

The obtained release dependencies were analyzed using the zero-, first-, and second- order kinetic models, as well as the Higuchi, Korsmeyer–Peppas, and Peppas–Sahlin models. The used equations have already been presented in previous works [17,30,31,53] and are shown in Figure 5. The program used for the calculations was Excel from Microsoft Office LTSC Standard 2021.

### 2.6. Statistical Analysis

The obtained data are presented as the arithmetic means from six measurements ± SDs (standard deviations). Statistical analysis of the viscosity measurements was carried out using Student’s *t*-test. The F1 formulation was the reference hydrogel. The confidence level was set at 95%. The least squares method was employed to determine the kinetic parameters, and the value of the correlation coefficient R^2^ allowed us to select the model that best described the release process of Bu from the tested hydrogels.

### 2.7. FTIR-ATR Measurements

The obtained hydrogels were dried at 6 °C. The preparations were ground in a mortar. The physical mixtures of all compositions, consisting of the pure polymers and Bu, were also ground in a mortar. Samples of the preparations, physical mixtures, and pure ingredients were studied using a spectrometer in ATR mode (Nicolet iS50, Thermo Scientific, Waltham, MA, USA). For each measurement, 32 scans were recorded at room temperature at a scanning speed of 65 scans per min. The FTIR-ATR spectra were obtained in the wavenumber range from 4000 to 400 cm^−1^, with a resolution of 4 cm^−1^.

### 2.8. DSC Measurements

A DSC study was carried out employing a differential scanning calorimeter (DSC 214 Polyma, Netzsch, Selb, Germany). Samples weighing 3–5 mg were introduced into dedicated aluminum crucibles covered with pierced lids. Thermograms of all formulations, their physical mixtures, and pure components were obtained in the temperature range of −10 to 300 °C at a heating rate of 5 °C/min under a nitrogen atmosphere. The gas flow rate was set at 25 mL/min.

## 3. Results and Discussion

### 3.1. Viscosity Study

The measured pH values of all compositions studied are presented in Table 2. The pH of formulation F1′ was slightly above neutral because it contained the salt of hyaluronic acid only. This compound, which is a salt of a strong base and a weak acid, dissociates in water into Na^+^ cations and polyanions, which are responsible for the obtained pH. The pH values of the F4′ and F5′ formulations were also close to neutral, because in addition to HA, the PVA-PVP mixture or PEG was added, which are non-ionic polymers (Figure 2). The lowest pH was obtained in the case of formulation F2′, doped with PA, which is an anionic polymer and responsible for lowering the pH of the preparation. The incorporation of AX in formulation F3′ resulted in a slight decrease in the pH. AX is a salt of a weak base, ammonia, which explains the reduction in the pH value of F3′. The results of the statistical analysis indicate differences in the pH among formulations F1′–F2′, F1′–F3′, F1′–F4′, and F1′–F5′. This means that the implementation of the synthetic polymer into the F2′–F5′ compositions affected their pH. The implementation of Bu into the formulations F1–F5 did not significantly change their pH, because a salt form of bupivacaine was used. This compound dissociates in water, releasing Cl^−^ anions and Bu^+^ cations (Figure 3b), which may react with other anions in the solution, changing its pH and even forming a new salt. The results of the statistical analysis revealed that the addition of the drug to formulations F1, F2, F3, and F5 influenced the pH of these hydrogels. The only exception was formulation F4, in which the pH did not depend on the presence of Bu. No differences in the pH between F4′ and F4 were found.

The dynamic viscosities of the hydrogel samples at 37 °C and room temperature (25 °C) are presented in Table 2. The viscosities at 37 °C were lower than the corresponding values at room temperature. This is consistent with the general rule that the viscosity decreases with increasing temperature. A comparison of the viscosity results at 25 °C and 37 °C, based on Student’s *t*-test, indicates differences among the viscosities of formulations F1′–F5′. The incorporation of the modifying polymer to the formulation increased the dynamic viscosity in all assessed systems. The viscosity of formulation F1′—containing only the HA polymer—was the lowest, while the viscosities of formulations F2′–F5′, with variable synthetic polymers and HA, were remarkably higher. The highest viscosity was recorded when AX was introduced into the hydrogel. Regarding the rheometric assessments of preparations F1–F5 containing Bu, a similar relationship was observed. The F1 formulation of HA and Bu only was the least viscous. However, the recorded viscosities of the bipolymeric systems with Bu were significantly higher. It may be concluded that the introduction of the modifying polymer to the hydrogel composition increased the viscosity of the formulations. The increases in viscosity may be attributed to the increased concentration of the entire content of macromolecules in the F2′–F5′ and F2–F5 formulations. However, the differences in the viscosity among the bipolymeric systems should be evaluated on the basis of the interactions between the main HA polymer and the additional polymers: PA, AX, PVA/PVP, and PEG.

The observed small increase in the viscosity of F2′ in comparison to that of F1′ may be related to the interaction between the HA chains and the chains of the additional polymer PA. Giubertoni et al. [54] revealed the interchain hydrogen bonds O=C–N–H∙∙∙O–C=O in aqueous hyaluronan. This suggests a similar bond formation between O=C–N–H from HA and the O–C=O group from PA and may explain the slight increase in the viscosity of F2′ after the addition of PA.

AX is a cationic compound, and the viscosity of the F3′ hydrogel containing AX was significantly higher than those of the other samples. This could be explained by the attraction of oppositely charged functional groups in the chains of HA and AX, i.e., carboxyl and cationic groups [55]. Regarding formulation F4′, the bond formation between HA and PVP, possibly via the carboxylic and hydroxyl groups, was reported by Lewandowska et al. [20]. The addition of the PVP-PVA mixture to HA blended improved the elastic properties of the preparation, which is due to the interaction between the macromolecules. The combination of these polymers reduces the formation of intramolecular hydrogen bonds within HA and promotes the formation of new hydrogen bonds between the chains. The FTIR spectra indicate that the OH groups and carboxylate groups from HA and the carbonyl group (C=O) from PVP were involved in bond formation. This explains the increased viscosity of formulations F4′ and F4 in comparison to the viscosity of hydrogels F1′ and F1, respectively. However, the viscosity of F4′ was lower than that of F3′. PVP is a non-ionic polymer. The hydrogel composed of PVP-PVA and HA have too low an ionic strength, which could have led to the mutual repulsion of the macromolecular chains and, consequently, to a decrease in the viscosity of hydrogel F4′ in comparison to that of F3′ [20]. Le-Deygen et al. [56] reported the interaction between HA and PEG. They found that oxygen atoms belonging to PEG had a large negative charge and were proton acceptors. Monosaccharides in the HA chains contained hydroxyl groups capable of forming hydrogen bonds as proton donors. Additionally, it was revealed that the addition of PEG to an HA hydrogel induced the compaction of the HA layer, confirming the bond formation between the polymers. This explains the increase in the viscosities of formulations F5′ and F5 in comparison to those of formulations F1′ and F1, respectively. Moreover, PEG is an amphiphilic polymer. It has hydroxyl groups in its structure. The addition of this compound to the HA-based hydrogel caused a significant decrease in the viscosity of the F5′ preparation in relation to the highest viscosity of F3′. This may be the result of the repulsion of the macromolecular chains, similar to the effect observed when PA was introduced into the system. In conclusion, the incorporation of the synthetic polymer to the HA-based hydrogel increased the viscosity of the carrier in the cases evaluated here. This is consistent with the data reported by Biswas et al. [57]. Mahmood et al. [2] reported in their work that the combination of a natural polymer with a synthetic polymer decreased the biodegradation and increased the durability of the resulting composition.

The comparison of the viscosities of the hydrogels containing Bu with the respective preparations without Bu (namely, F1′ and F1, F2′ and F2, etc.) showed that the addition of the drug to the compositions changed their viscosities. It is interesting to note that the incorporation of Bu to formulations F1-F3, containing only ionic polymer HA, or with ionic additive polymers PA or AX, decreased the viscosity in comparison to the viscosities of the compositions without Bu, F1′–F3′. In contrast, the presence of Bu in formulations F4 and F5, supplemented with non-ionic polymers PVA-PVP or PEG increased the viscosities of the hydrogels. This may be related to the ionic interactions of Bu with polymers and should be further studied.

### 3.2. Release Study

The dissolution curves of Bu from formulations F1–F5 are presented in Figure 4. The drug release test was carried out in phosphate-buffered solution with a pH of 6.8, which reflects the environment of inflamed synovial fluid [51].

It was found that, after 8 h of testing, the highest amount of drug released, 92.4 ± 4.6%, was from formulation F1, which did not contain the synthetic polymer. The amount of Bu released after the same amount of time from the other hydrogels, which were doped with synthetic polymers, was lower. This suggests that the addition of synthetic polymers to hydrogels may prolong the drug release. The lowest concentration of Bu, 77.0 ± 2.3%, was obtained in the acceptor fluid when the drug was released from formulation F3, which was doped with AX. This indicates that the optimal synthetic polymer that enabled a prolonged drug release was AX. These results correlate well with those of the viscosity study. The viscosities of hydrogels F3 and F3′, which were doped with AX, were the highest, suggesting that the drug transport to the acceptor medium was hindered due to the densely packed polymer network. These obtained results are consistent with previous outcomes [17,18]. In studies on the release of 5-ASA (5-aminosalicylic acid, mesalazine) from formulations based on pectin doped with the same synthetic polymers as in the present work, it was revealed that the addition of synthetic polymers to the preparation may prolong the drug release. However, when 5-ASA was released from pectin-based preparations, the lowest concentration of the drug in the acceptor medium was obtained after 8 h of measurement in the case of the composition doped with PA [17,18]. Patel et al. [58] studied the influence of polymer viscosity on drug release and observed that the viscosity of HPMC had a dominant role as a controlling factor in the drug release kinetics. Kottke et al. [59] found that the viscosity affected the drug’s solubility and the dissolution rate, which directly affected its bioavailability. Highly viscous formulations can hinder drug release, which affects the drug’s absorption and overall efficacy.

### 3.3. Release Curve Comparison

The obtained dissolution profiles of Bu from hydrogels F1-F5 were compared based the difference factor f_1_ and the similarity factor f_2_ [60]. According to regulations, a value of f_1_ below 15 and a value of f_2_ above 50 indicate similarity between the compared profiles. The parameters f_1_ and f_2_, presented in Table 3, were calculated by comparing the Bu release profile from formulation F1, which did not contain a synthetic polymer, to the Bu dissolution curves from formulations F2–F5, which were doped with the synthetic polymers.

In the comparison of the Bu dissolution profile from F1 with the Bu release curve from hydrogel F3, it was observed that the value of f_1_ was 18.2 (above 15) and f_2_ was 47.2 (below 50), indicating a discrepancy. The observed differences are marked in Table 3 with an asterisk (*), indicating that only the incorporation of AX to the carrier influenced the drug release. This correlates well with the results of the viscosity study, which revealed that the F3 hydrogel had the highest viscosity, while the F1 hydrogel showed the lowest viscosity. Consequently, we observed discrepancies between the Bu release profiles from the hydrogel F1 and the hydrogel F3. In conclusion, it can be stated that the introduction of AX into the HA-based matrix affected the release of Bu from the hydrogel. The comparison of the present results with the results obtained from the study of 5-ASA release from pectin beads doped with the same synthetic polymers as in the presented investigation is of interest. It was reported that the incorporation of AX and PA into the pectin-based carrier did not have strong influence on the release behavior of 5-ASA, although the addition of PVP and PEG changed the dissolution of the drug from the matrix [17].

### 3.4. Kinetic Analysis

The experimental points of Bu dissolution from hydrogels F1-F5 were fitted to theoretical curves using the least squares method. The release rate constants k, the half-release time t_0_._5_, and the correlation coefficients R^2^ based on the zero-, first-, second-order kinetic models, as well as the Higuchi, Korsmeyer–Peppas, and Peppas–Sahlin models, were derived from this analysis. As an example, the curve-fitted data of Bu release from hydrogel F1 are presented in Figure 5. The Korsmeyer–Peppas and Peppas–Sahlin equations were applied to the first 60% of the drug release [53].

The calculated kinetic parameters are shown in Table 4. The results of the analysis of the correlation coefficients indicate that, in all cases, the release kinetics were most appropriately described by the Higuchi model, with R^2^ values ranging from 0.96 ± 0.2 to 0.99 ± 0.01. The Korsmeyer–Peppas model was also suitable, with R^2^ values ranging from 0.85 ± 0.2 to 0.99 ± 0.01. Moreover, the release rates of the drug from F4 and F5 were also defined by the first-order kinetics, with a correlation coefficient of 0.99 ± 0.01.

It is worth noting that the release rate constant derived from the Higuchi model, 5.3 ± 0.1 mg × min^−1/2^, was the highest in the case of Bu released from F1, which did not contain the synthetic polymer. The lowest value of k_H_ (4.4 ± 0.2 mg × min^−1/2^) was calculated when the substance was released from F3, which was doped with AX. According to the Korsmeyer–Peppas equation, the same trend was observed: the highest value of the release rate constant (5.8 ± 0.1) × 10^−2^ min^–N^ was obtained when Bu was released from formulation F1, whereas the lowest value of k_K-P_ = (3.0 ± 0.2) × 10^−2^ min^–N^ was derived in the case of the drug dissolution from hydrogel F3. Additionally, a similar tendency was also found from the other models, confirming that Bu was released the fastest from the preparation that did not contain the synthetic polymer, whereas it was released the slowest from the hydrogel incorporating AX. It should be mentioned that the presented kinetic results are in good agreement with the data presented in our previous work on the release of 5-ASA from a pectin carrier doped with the same macromolecules. The mass transfer was the fastest from the formulation that did not contain the second polymer [17]. The results of the analysis of the half-release time based on the Higuchi model indicate that half of the drug was released within 134 ± 7.0 min from F1, which was the shortest half release time. The highest value of t_0_._5_ 199.5 ± 17.6 min was obtained from F3. The same trend was found according to the other models. It can be concluded that the incorporation of the second macromolecule to the carrier, in particular AX, reduced the drug release rate while extending the half-release time from the matrix. The parameter n, characterizing the drug transport mechanism from the matrix to the acceptor fluid, was calculated using the Korsmeyer–Peppas equation. It was found that in all release tests carried out in this study, the value of n was above 0.5, indicating that the drug was transported from the carrier to the medium via anomalous transport [61,62]. Ritger and Peppas [63] explained that the release parameter n provided important information on the mass transport from the polymeric matrix. According to these authors, anomalous transport, that is, non-Fickian behavior, is a coupling of diffusion and relaxation phenomena [64]. In addition, the obtained drug dissolution profiles may have also been influenced by other factors, such as the crosslinking density, swelling/deswelling behavior, and the mesh size of the hydrogel network. Moreover, the formation of a polymer network may lead to irregularities and, consequently, the pore size may be heterogeneous, resulting in variability among the dissolution profiles [65].

Babu at al. [53] reported that the parameter n in the Korsmeyer–Peppas equation depends on the solvent diffusion rate (R_diff_) and the polymer chain relaxation rate (R_relax_). The value of n obtained in the present work ranged from 0.51 ± 0.02 to 0.58 ± 0.02. According to the interpretation of Babu et al. [53], a value of n between 0.45 and 0.89 indicates an anomalous (non-Fickian) diffusion mechanism of drug transport, where the diffusion and the relaxation rates are similar (R_diff_ ≈ R_relax_). The Peppas–Sahlin model contains two release rate constants: k_1P-S_, the release rate constant for the Fickian contribution to the drug release, and k_2P-S_, the release rate constant for the polymeric chain relaxation (Case II transport) contribution to the drug release. The first term, $k_{1P-S}t^{n\prime }$, represents the Fickian diffusion influence, and the second term, $k_{2P-S}t^{2n\prime }$, indicates the role of polymeric system relaxation. Additionally, researchers have revealed that in cases where the value of $k_{1P-S}$. is higher than $k_{2P-S}$, Fickian diffusion is the more dominant transport mechanism of the drug compared to polymer relaxation and swelling in such carriers. However, when the value of $k_{2P-S}$ is higher than $k_{1P-S}$, the levels of polymer relaxation and matrix swelling are comparable, supporting the tendency for drug transport to occur via non-Fickian kinetics. In the present work, the values of $k_{2P-S}$ were higher than the values of $k_{1P-S},$ indicating the predominance of anomalous transport over Fickian diffusion. This is consistent with the values of the exponent n in the Korsmeyer–Peppas equation (above 0.5), which also indicated anomalous transport. However, the values of the parameter n’ in the Peppas–Sahlin model were about 0.3, suggesting Fickian diffusion. Colpo et al. [66] studied the release of lidocaine from double-setting α-tricalcium phosphate cement and obtained values of n of about 0.30 and 0.33 using Korsmeyer–Peppas model; however, the values of n’ calculated using the Peppas–Sahlin model were slightly above (0.51 and 0.54). It was concluded that the release of the drug first followed Fickian diffusion and then changed into polymeric chain relaxation. In the present work, the drug transport may have also been a combination of Fickian diffusion and polymeric chain relaxation. The burst effect at the beginning of the release was related to drug transport according to Fickian diffusion, but it changed during the slower release. The explanation of the drug release mechanism is very important for medical professionals, especially anesthesiologists, because it broadens the range of treatment choices, enabling a balance between an initially intensive effect and a prolonged analgesic effect [66].

From a practical point of view, the variability in the half-release times indicates that F3 was the most suitable formulation for the prolonged release of Bu, whereas the interactions in F1 were less pronounced, making it release the drug the fastest in the acceptor medium. The results obtained from the kinetic study correlate well with the viscosity data. The viscosity of the F3 hydrogel was the highest, which strongly influenced the transport of the drug from the interior of the matrix to the acceptor medium. This resulted in the slowest drug release from this formulation. AX was the optimal polymer and most likely to ensure the prolonged release of the drug from the doped hydrogel. Bayer [67] reported that the HA matrix acts as a tunable carrier for drug release. The structure of HA ensures its physical crosslinking and conjugation with other macromolecules, leading to the formation of controlled or sustained drug release systems.

### 3.5. FTIR-ATR Study

**Figure 6:**
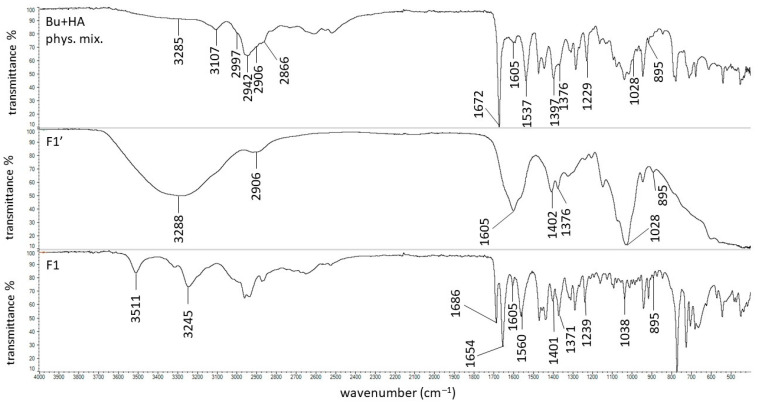
The FTIR-ATR spectra of the physical mixture of Bu and HA, the F1′ formulation, and the F1 formulation.

The FTIR spectrum of Bu was presented in our previous work, where all characteristic bands were found at 3106, 2997, 2942, 2866, 1672, 1537, 1397, and 1229 cm^−1^ [68]. The FTIR spectrum of HA was also presented in our previous work; the main signals assigned with HA were present at 3285, 2906, 1605, 1402, 1376, 1028, and 895 cm^−1^ [69]. The spectra of the physical mixture of Bu and HA, formulation F1′, and formulation F1 were recorded in the present study and are shown in Figure 6.

**Figure 7:**
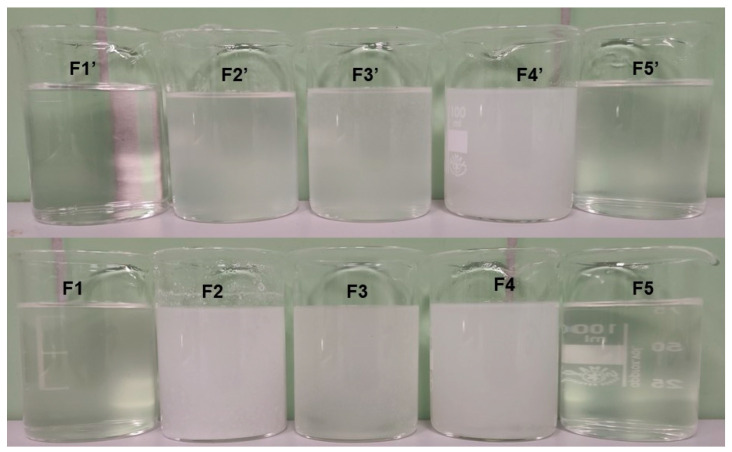
The visualization of prepared hydrogels.

**Figure 8:**
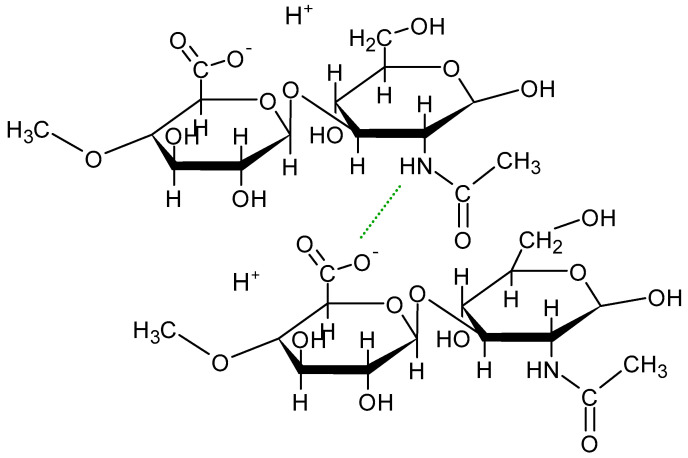
The hydrogen bond formation (green line) in the HA macromolecule.

The maxima attributed to Bu and HA were observed on the spectrum of the physical mixture of these ingredients, although the peaks coming from HA were weaker or overlapped by stronger signals belonging to Bu. On the FTIR-ATR spectrum of the F1′ formulation, composed of HA and water, the bands of HA were found. The band at 3288 cm^−1^ broadened, suggesting the formation of a hydrogen bond. This is consistent with the work of Larrañeta et al. [36], in which it was postulated that covalent bonds do not exist in HA hydrogels, although hydrogen bonds may occur between the macromolecules or between the macromolecules and water. The obtained hydrogel F1′, presented in Figure 7, was transparent. This may confirm the absence of covalent bonds. Additionally, Giubertoni et al. [54] postulated the direct interchained hydrogen bond formation of O=C–N–H∙∙∙O–C=O in the HA macromolecule, meaning that in the present work, similar bond formation may have occurred. The hydrogen bond formation is presented in Figure 8.

**Figure 9:**
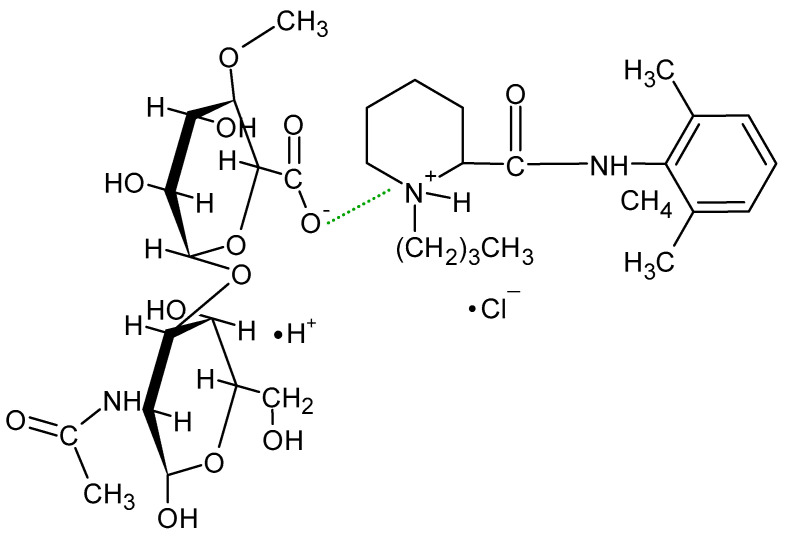
The interaction (green line) between Bu and HA units.

The FTIR-ATR spectrum of the F1 formulation is very interesting because a new peak appeared at 3511 cm^−1^, which was observable neither on the spectrum of pure Bu nor on the spectrum of the physical mixture of Bu and HA. Niederwanger et al. [70] reported that Bu occurs in five crystalline forms: four anhydrous forms (A, B, C, and D) and a monohydrate. The IR spectra of these five forms indicate that signals at 3517 cm^−1^ and at 3434 cm^−1^ were present only on the spectrum of the monohydrate form and related to O-H stretching vibrations. This may explain the presence of the band at 3511 cm^−1^ on the spectrum of F1. According to de Castro et al. [71], the band at 3511 cm^−1^ is related to the ammonium salt because, in this study, Bu occurred in the form of hydrochloride. The next observed signal at 3245 cm^−1^ may be due to the stretching of the hydrogen-bonded N–H group of the mono-substituted amides O=C–N–H belonging to Bu as well as to HA [69,71,72]. However, an investigation of the relationship between PA and verapamil hydrochloride indicated that anionic polymers, such as PA, will form a salt with the –NH^+^ group of the drug [73]. This suggests that, in the present work, salt may also be formed between the –COO^−^ group of HA and –NH^+^ group belonging to Bu. This may explain the slight turbidity of the F1 hydrogel (Figure 7) in comparison to the F1′ formulation, which was Bu-free. Moreover, the interaction between HA and Bu may explain the increase in the viscosity of the F1 hydrogel after the introduction of Bu. The proposed interaction is presented in Figure 9. The maxima at 3511 cm^−1^ and 3245 cm^−1^ were also observed in the spectra of formulations F2–F5, indicating the interaction between Bu and HA in these formulations. The viscosity of F1–F3 increased after the Bu was incorporated, confirming the possibility of an interaction between HA and Bu in these formulations, although the addition of Bu to the F4–F5 hydrogels decreased their viscosities.

**Figure 10:**
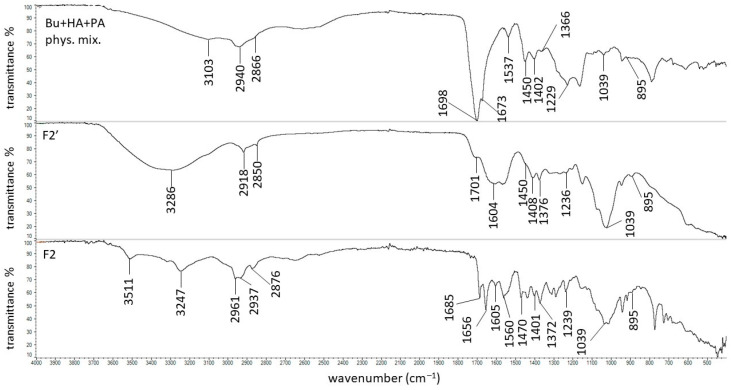
The FTIR-ATR spectra of the physical mixture of Bu, HA, and PA; the F2′ formulation; and the F2 formulation.

Formulations F2-F5 were doped with synthetic polymers, such as PA, AX, PVP, and PEG, and the FTIR spectra of these compounds were recorded. The distinctive peaks coming from PA occurred at 2940, 1698, 1450, 1402, and 1229 cm^−1^, correlating well with the data in the literature [69]. All of the characteristic bands of Bu, HA, and PA were present on the spectrum of the physical mixture of these compounds (Figure 10), although some of them were very weak or overlapped by other strong signals.

**Figure 11:**
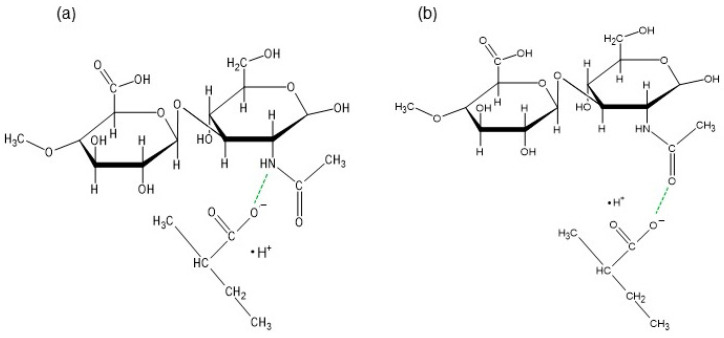
The interaction (green line) between HA units and PA units, (**a**) between –NH group from HA and –COO^−^ group of PA (**b**) between –C=O group from HA and –COO^−^ group of PA.

The wide maximum at 3286 cm^−1^ on the spectrum of F2′ (Figure 10) is similar to the band on the spectrum of the F1′ formulation and confirms the existence of the hydrogen bond in the hydrogel between the polymer chains or between the macromolecules and the water. It should be mentioned that in the F2′ spectrum, the peak at 1701 cm^−1^ corresponds to the strong signal of PA at 1698 cm^−1^ in the pure PA spectrum, which comes from its carboxyl group. A signal at 1604 cm^−1^, which belongs to HA, was also found. However, the peak was weaker in comparison to the band on the spectrum of pure HA. According to the study on the interaction between PA and verapamil hydrochloride, a salt was formed between the –COO^−^ group of PA and the –NH^+^ group of the drug [73]. This suggests that a similar bond may have also formed between the –COO^−^ group of PA and the –NH^+^ group belonging to the HA macromolecule. Additionally, the direct interchained hydrogen bond formation of O=C–N–H∙∙∙O–C=O in the HA macromolecule was also reported in [54]. This suggests that, in the present work, the possibility of bond formation between the O=C–N–H group from HA and the O–C=O group from PA may also exist (Figure 11a). On the other hand, an interaction between the hydroxyl group from the –COOH belonging to PA and the oxygen lone pair from the –C=O group coming from PVP was observed by Song et al. [74]. This suggests that, in the present work, a similar interaction was possible between the –COOH group from PA and the –C=O group from HA, which could explain the presence of the weaker signals of PA at 1698 cm^−1^ and HA at 1604 cm^−1^ (Figure 11b). The picture of the hydrogel F2′ presented in Figure 7 confirms that the incorporation of PA affected the HA-based preparation, which became turbid. Additionally, the bond formation between these polymers may be responsible for the increase in the viscosity of F2′ in comparison to that of F1′.

**Figure 12:**
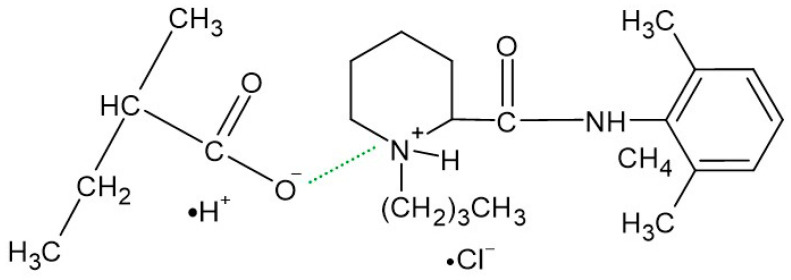
The interaction (green line) between Bu and PA units.

The incorporation of Bu into the formulation consisting of HA, PA, and water affected the spectrum of F2 (Figure 10). A new band appeared at 3511 cm^−1^, which is assigned to O–H bond stretching in Bu monohydrate. Additionally, a new maximum at 3247 cm^−1^, corresponding to the N–H stretching, was also present in the spectrum of F2. This suggests that, as in formulation F1, a salt may be formed between HA and Bu [71]. Moreover, in addition to HA, the second polymer (PA) present in formulation F2 and containing an O–C=O group, may have also interacted with Bu (Figure 12). The signals at 1698 cm^−1^ and 1450 cm^−1^ in the spectrum of the physical mixture of Bu, HA, and PA, which originated from PA were shifted to 1685 cm^−1^ and 1470 cm^−1^, respectively, in the spectrum of F2. The amide carbonyl stretching band (C=O) of Bu observed in the spectrum of the physical mixture of Bu, HA, and PA at 1673 cm^−1^ was observed in the spectrum of F2 at 1656 cm^−1^. Simultaneously, the peak of Bu at 1537 cm^−1^ in the spectrum of the physical mixture was found in the F2 spectrum at 1560 cm^−1^. These small shifts may also indicate an interaction between PA and Bu. This interaction was also reflected in the greater turbidity of the F2 preparation compared to the F2′ formulation (Figure 7). Negatively charged PA may react with positively charged Bu to form a salt. This reaction may also explain the viscosity increase in F2.

**Figure 13:**
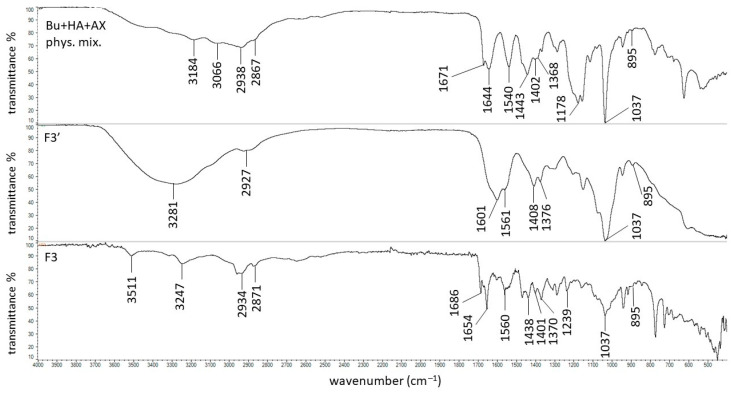
The FTIR-ATR spectra of the physical mixture of Bu, HA, and AX; the F3′ formulation; and the F3 formulation.

The FTIR spectra of pure AX showed the main maxima at 1640, 1544, 1440, 1388, and 1176 cm^−1^, which is in good agreement with previous results [17]. All these peaks were present on the FTIR spectrum of the physical mixture of Bu, HA, and AX. This spectrum and the spectra of formulations F3′ and F3 are shown in Figure 13.

**Figure 14:**
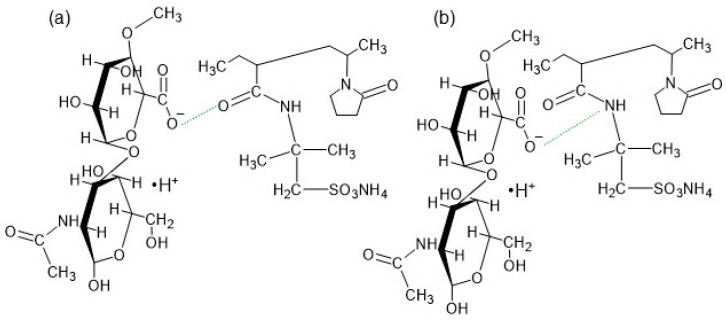
The interactions (green line) between HA and AX units: (**a**) between the –C=O group of AX and the –COO^−^ group of HA, and (**b**) between the –COO^−^ group of HA and the –NH group of AX.

In the spectrum of the F3′ formulation, none of the mentioned bands belonging to AX occurred, apart from the weak signal at 1561 cm^−1^. This may be related to the interaction between the polymer molecules. The same results were obtained in a previous FTIR study on amidated pectin doped with AX and incorporated with mesalazine [17,18]. It was postulated that the absence of these maxima may indicate an interaction between the carbonyl groups of AX and the carboxyl groups of the amidated pectin or even the mesalazine. In the present investigation, it was possible that the same interaction between the –C=O group of AX and the –COO^−^ group of HA occurred because these characteristic peaks belonging to AX were neither on the F3′ spectrum nor on the F3 spectrum (Figure 14a). Additionally, apart from the interaction between the carbonyl groups of AX and the carboxyl groups of HA, bond formation between the –COO^−^ group of HA and the –NH group belonging to AX was also possible (Figure 14b).

The introduction of AX to the HA-based preparation also resulted in the turbidity of the resulting composition, which can be attributed to the interaction between the polymers. Additionally, the possibility of two types of bond formation (Figure 14) between HA and AX may explain the highest viscosity of the formulation doped with AX. Moreover, the addition of the drug to the F3 hydrogel caused even greater turbidity, suggesting the presence of some other interaction (Figure 7). The broad band at 3280 cm^−1^ observed in the spectrum of F3′ (Figure 13) indicates hydrogen bond formation between the polymer molecules or between the polymers and the water, similar to that observed in the spectra of the F1′ and F2′ formulations. It should be noted that in the spectrum of F3, the maxima were found at 1686 cm^−1^ and 1654 cm^−1^. Both of these bands were also visible in the F2 and F1 spectra. This may indicate bond formation between Bu and HA, since both of these compounds were present in formulations F1–F3.

**Figure 15:**
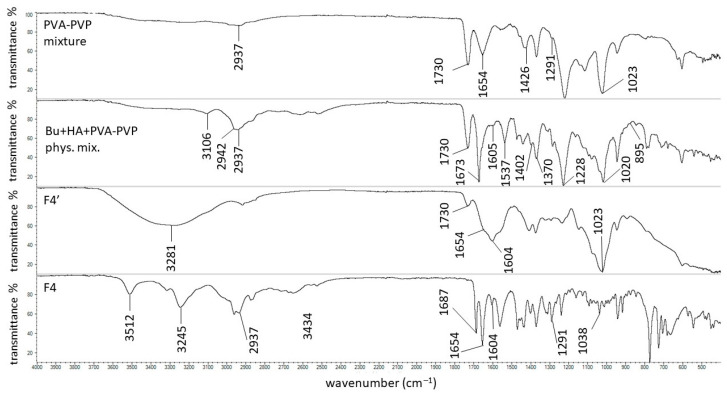
The FTIR-ATR spectra of the physical mixture of Bu, HA, and PVA-PVP; the F4′ formulation; and the F4 formulation.

The FTIR-ATR spectrum of the PVA-PVP mixture is presented in Figure 15. The characteristic bands at 3434 cm^−1^ (belonging to the O-H stretching vibration), 2937 cm^−1^ (attributed to the C-H asymmetric stretching vibration), 1730 cm^−1^ (from the C=O stretching vibration of PVA), 1654 cm^−1^ (belonging to the C=O stretching vibration of PVP), 1426 cm^−1^ (assigned to the CH_2_ bending vibration), and 1291 cm^−1^ and 1023 cm^−1^ (related to the C-N vibrations) were found and correlate well with the data from the literature [75,76]. All the maxima, as well as the signals coming from Bu and HA, were present in the spectrum of the physical mixture of Bu, HA, and PVA-PVP.

**Figure 16:**
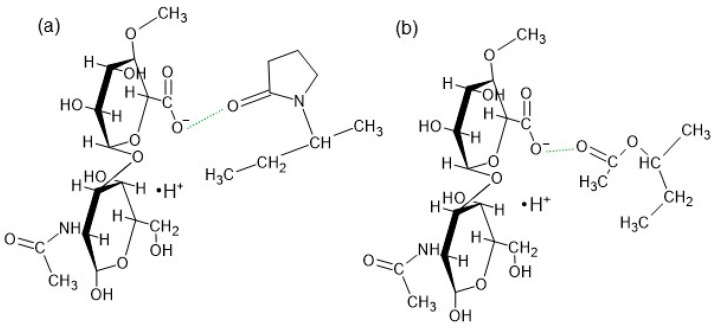
The interactions (green line) between HA and (**a**) PVP and (**b**) PVA.

A very broad maximum occurred at 3281 cm^−1^ in the spectrum of F4′, assigned to the presence of water. Rahma et al. [75] explained that the wide signal in this region indicates the possibility of hydrogen bond formation between the –C=O groups of pyrrolidone rings from PVP. Similar to the F1′ formulation, hydrogen bonds between the HA chains may also exist. However, Lewandowska et al. [20] revealed an interaction between HA and PVP. The carboxylate groups in HA and the carbonyl groups from the pyrrolidone rings in PVP generated new hydrogen bonds (Figure 16a). In the spectrum of F4′, the signal at 1730 cm^−1^ from PVA was reduced, the band at 1654 cm^−1^ from PVP was very weak, while the signal at 1604 cm^−1^ from HA remained. These observations may indicate that not all groups took part in the hydrogen bond formation. The interaction of the –C=O groups from PVP and PVA and the –COO^−^ groups from HA reduced the intramolecular hydrogen bond formation in the HA chains. Finally, the mechanical properties and the viscosity behavior of the obtained carrier changed. This interaction explains the turbidity of formulation F4′ presented in Figure 7. This bond formation may also explain the increase in the viscosity of hydrogel F4′ in comparison to F1′, which is attributed to the introduction of the PVP-PVA mixture.

**Figure 17:**
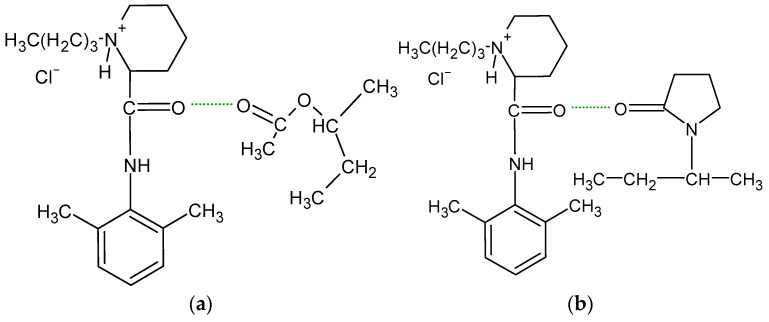
The interaction (green line) between Bu and (**a**) PVA and (**b**) PVP.

The FTIR-ATR spectrum of the F4 formulation shows that the band at 1730 cm^−1^ shifted toward a lower wavenumber and appeared at 1687 cm^−1^. Additionally, the signal at 1023 cm^−1^ was reduced and appeared at 1038 cm^−1^. These discrepancies could be associated with the incorporation of Bu into the formulation. Rahim et al. [77] studied the possibility of interaction between C=O groups. It was revealed that the lone electron pair (n) located on the oxygen atom of the carbonyl group can be delocalized over a π* orbital of another carbonyl group. It was observed that such interactions were present in 3D structures of polyesters, peptides, and proteins. According to this study, the observed changes in the F4 spectra may have been caused by the interaction between the C=O groups from PVA and Bu (Figure 17a) and between the C=O groups from PVP and Bu (Figure 17b). The picture of formulation F4 in Figure 7 indicates that the hydrogel was milky, although it is difficult to clearly state whether it was the result of the interaction between the polymers only, as observed in the F4′ formulation, or whether it was also influenced by the introduction of the drug into the formulation. However, the incorporation of the drug resulted in a decreased viscosity of F4, suggesting that an interaction between Bu and PVP-PVA did not occur.

**Figure 18:**
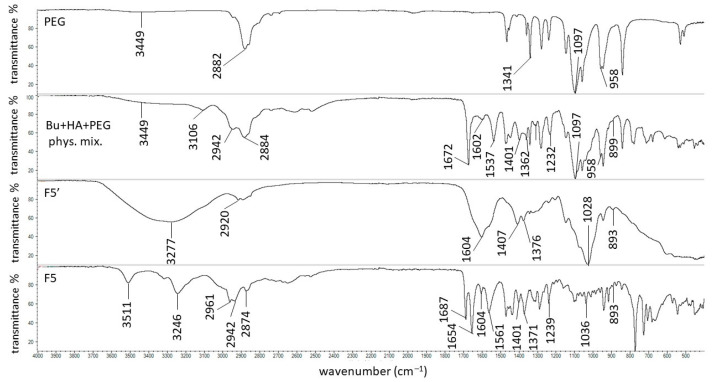
The FTIR-ATR spectra of the physical mixture of Bu, HA, and PEG; the F5′ formulation; and the F5 formulation.

The FTIR-ATR spectrum of pure PEG, shown in Figure 18, exhibits typical signals at 3449 cm^−1^ (attributed to O–H stretching vibrations), 2882 cm^−1^ and 958 cm^−1^ (attributed to CH_2_ asymmetric stretching vibrations), 1097 cm^−1^ (related to C−O stretching vibrations), and 1341 cm^−1^ (antisymmetric stretching). These results correlate well with the results of the study by Zhang et al. [78]. All these peaks were observed in the spectrum of the physical mixture of Bu, HA, and PEG. The representative bands of Bu and HA were also found. The broad maximum at 3277 cm^−1^ visible on the FTIR-ATR spectrum of the F5′ formulation indicates hydrogen bond formation between the same polymer, between a macromolecule and water, or between chains belonging to PEG and HA. The interaction between PEG and HA was studied by Le-Deygen et al. [56], who found that oxygen atoms belonging to PEG molecules were proton acceptors coming from the hydroxyl group of HA. These weak hydrogen bonds resulted in the transparency of preparation F5′, similar to preparation F1′ (Figure 7) and may also explain the increase in the viscosity of F5′. No interaction between PEG and Bu was found. This is consistent with the picture of the F5′ and F5 preparations in Figure 7. The appearance of hydrogel F5 after the introduction of Bu remained unchanged in comparison to F5′.

### 3.6. DSC Investigation

**Figure 19:**
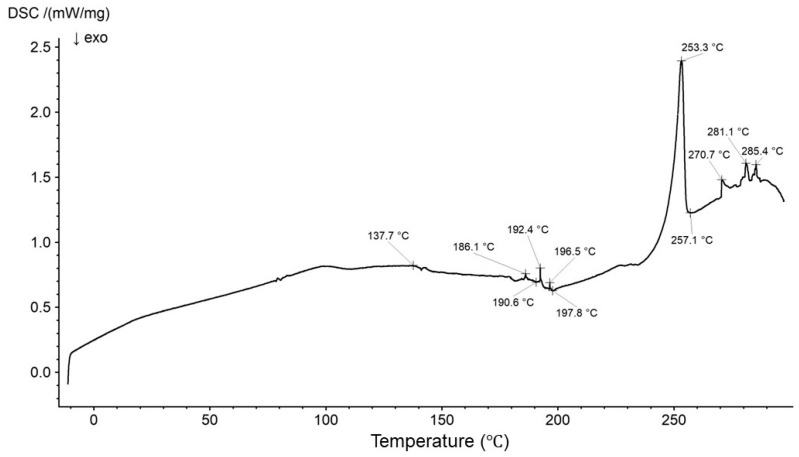
The thermogram of bupivacaine hydrochloride (Bu).

A thermogram of HA was recorded, and the main maxima observed at 77.5 °C, 148.3 °C, 231.6 °C, and 244.7 °C are in good correlation with the signals presented in our previous study [69]. The recorded thermogram of Bu is shown in Figure 19.

**Figure 20:**
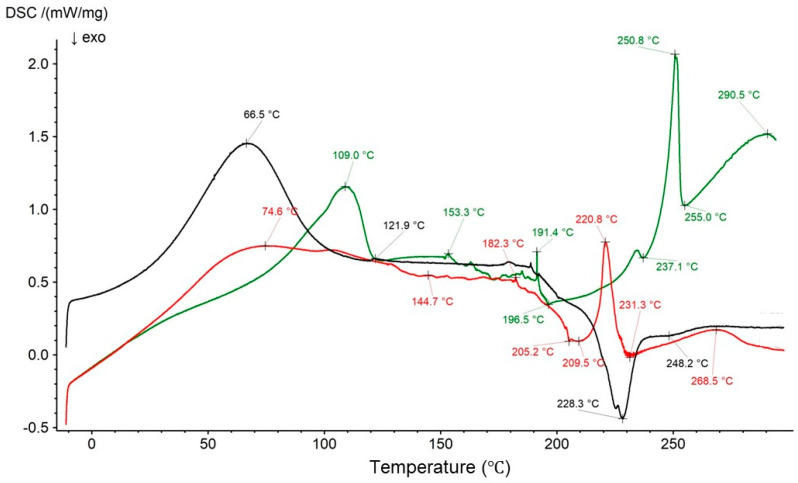
The thermograms of the physical mixture of HA and Bu (red), formulation F1′ (black), and formulation F1 (green).

A thermogram of the physical mixture of HA and Bu with the thermal curves of formulations F1′ and F1 is presented in Figure 20.

The thermogram of the physical mixture of HA and Bu shows an endotherm for HA at 74.6 °C and exotherms for HA at 205.2 °C and 209.5 °C. These maxima were slightly shifted in comparison to the thermogram of pure HA due to the introduction of the second component into the system—Bu [69]. The characteristic peak of Bu was observed in the thermogram of the physical mixture of HA and Bu at 220.8 °C. However, in the thermogram of pure Bu (Figure 19), it was present at 253.3 °C. The small endotherms belonging to Bu from 186.1 °C to 196.5 °C were found from ca. 144.7 °C to 182.3 °C, whereas the signals from 270.7 °C to 285.4 °C appeared as one wide maximum at 268.5 °C on the thermogram of the physical mixture of HA and Bu. They are likely overlapped by the thermogram of HA. In the thermogram of F1′, two endotherms at 66.5 °C and 121.9 °C and two exotherms at 228.3 °C and 248.2 °C were observed. These maxima are in good correlation with the peaks in the pure HA thermogram because formulation F1′ consisted of HA and water only. The implementation of Bu into the formulation caused the appearance of Bu peaks from 153.3 °C to 191.4 °C. A sharp Bu endotherm appeared at 250.8 °C with an exotherm at 255.0 °C. The endotherms from 270.7 °C to 285.4 °C turned into one endotherm at 290.5 °C. The endotherm belonging to HA was shifted to 109.0 °C in the thermogram of F1; simultaneously, the HA exotherms were found at 196.5 °C and 237.1 °C. These discrepancies may suggest an interaction between HA and Bu, which was postulated in the FTIR-ATR studies.

**Figure 21:**
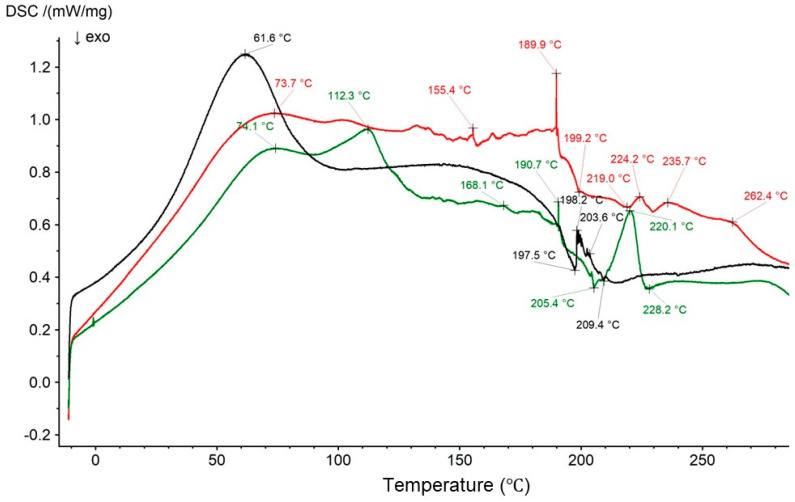
The thermograms of the physical mixture of HA, Bu, and PA (red); formulation F2′ (black); and formulation F2 (green).

The thermograms of the physical mixture of Bu, HA, and PA and the curves of formulations F2′ and F2 are presented in Figure 21. According to our previous work, the main peaks of PA were present at 62.4 °C and 221.5 °C [17]. All the main peaks belonging to the F2 ingredients were found in the F2 physical mixture thermogram. However, in the thermogram of the F2′ formulation, which did not contain the drug, the PA endotherm at 221.5 °C disappeared, whereas the second endotherm at 61.6 °C was present. Moreover, a new set of sharp maxima appeared between 198.2 °C and 203.6 °C, and it was difficult to attribute their presence to HA or to PA. This suggests a bond formation between the polymers, which was also found in the FTIR-ATR study. The exotherms at 197.5 °C and 209.4 °C may be assigned to HA. The endotherm of HA at 69.0 °C was overlapped by the strong signal from PA at 61.6 °C, and the second endotherm of HA at 124.2 °C was not visible because it was very weak. It is interesting to note that the addition of Bu to the formulation caused the appearance of a new endotherm at 112.3 °C on the thermogram of F2 and that the sharp signal belonging to Bu at 253.3 °C disappeared. The set of endotherms of Bu from 186.1 °C to 196.5 °C were weaker and present in the range of ca. 168.1–190.7 °C. These observations indicate a bond formation between Bu and the polymers. This correlates well with a study reported by Elkheshen et al. [73] and with the results from FTIR-ATR measurements. As mentioned in the discussion, the carboxyl group of PA may interact with the –NH^+^ group of the drug, which was verapamil hydrochloride. This means that, in the presented work, the carboxyl group belonging to PA or HA may also affect the –NH^+^ group of bupivacaine hydrochloride.

**Figure 22:**
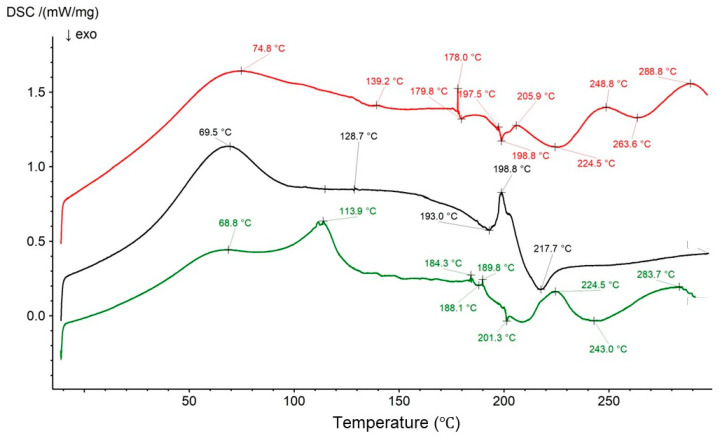
The thermograms of physical mixture of HA, Bu, and AX (red); formulation F3′ (black); and formulation F3 (green).

The thermogram of the physical mixture of Bu, HA and AX, presented in Figure 22, indicates all peaks assigned to these components, although some of the maxima are invisible because they are overlapped by others. For example, the peak at 74.8 °C may belong to HA or AX, and the peak at 248.8 °C may come from AX or Bu. The exotherm of pure HA at 228.5 °C was located in the thermogram of the physical mixture of Bu, HA, and AX at 224.5 °C, whereas the second exotherm of pure HA at 246.7 °C disappeared on the curve of the physical mixture. It should be noted that in the thermogram of the F3′ formulation composed of HA, AX, and water, some maxima did not exist. The endotherm attributed to AX at 248.8 °C was not found; simultaneously, the exotherm of HA at 246.7 °C was not present in the F3′ plot. Moreover, a new exotherm appeared at 193.0 °C, and a new endotherm was observed at 198.8 °C. These results indicate that similar bond formations between the polymers occurred as observed in formulation F2′. AX contains in its structure a –C=O group, like PA, and a –NH group, like HA, meaning that both of these groups may interact with the –C=O and –NH group of HA. The incorporation of Bu into the formulation changed the thermogram of F3 in comparison to the curve of F3′. It was revealed that a new peak appeared at 113.9 °C, similar to the thermogram of F2 (at 112.3 °C). Neither the strong endotherm of Bu at 253.3 °C nor the characteristic peak of AX at 248.8 °C was observed. The obtained results confirm the bond formation between Bu and AX, but an interaction between the drug and HA was also possible.

**Figure 23:**
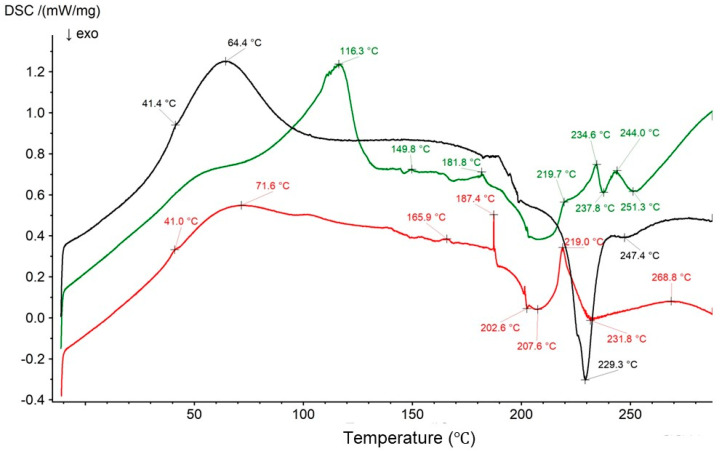
The thermograms of the physical mixture of HA, Bu, and PVA-PVP (red); formulation F4 (black); and formulation F4 (green).

The thermogram of the polymer mixture of PVA-PVP had only one endotherm at 49.2 °C. This signal was present on the curve of the physical mixture of Bu, HA, and PVA-PVP at 41.0 °C (Figure 23). The endotherm of HA was found at 71.6 °C, and two weak exotherms were identified at 202.6 °C and 207.6 °C. The series of Bu endotherms were present from ca. 165.9 °C to 187.4 °C. The main endotherm was observed at 219.0 °C, with a weak exotherm at 231.8 °C. A set of peaks between 270.7 °C and 285.4 °C was observed as one wide maximum at 268.8 °C. It can be concluded that all the characteristic peaks of the ingredients of the mixture were found. It is worth noting that in the thermogram of the F4′ formulation (Figure 22) composed of HA, the PVA-PVP mixture, and water, nothing of interest was found. The maxima belonging to HA were observed at 64.4 °C, 229.3 °C, and 247.4 °C. The very weak peak of the PVA-PVP mixture was present at 41.4 °C. It was difficult to find strong evidence confirming the interaction between HA and PVA-PVP using the DSC study only, although the FTIR study, viscosity investigations, and turbidity observations suggest an interaction between HA and PVP-PVA. According to the data in the literature, a hydrogen bond may be also formed [36,54].

It should be noted that in the thermogram of the F4 formulation, a minor peak appeared at 116.3 °C. Similarly, in the case of formulation F3, a new signal was observed at 113.9 °C, while formulation F2 showed a peak at 112.3 °C and formulation F1 showed a peak at 109.0 °C. This may confirm the bond formation between Bu and HA, since both compounds were present in formulations F1-F4. Moreover, another strange signal was present at 244.0 °C. There were no maxima in this region belonging to any component, apart from the endotherm of Bu, which was present at 234.6 °C. This observation may indicate an interaction between Bu and the PVA-PVP mixture, because PVP and PVA each contain a carbonyl group in its structure.

**Figure 24:**
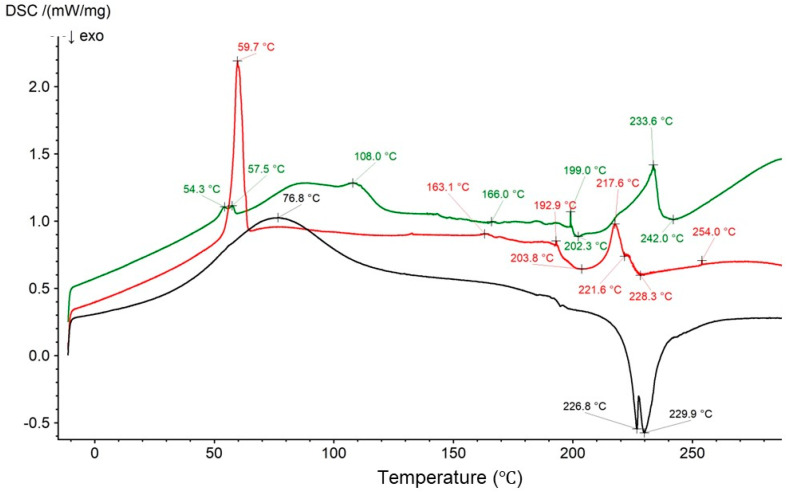
The thermograms of the physical mixture of HA, Bu, and PEG (red); formulation F5′ (black); and formulation F5 (green).

Figure 24 presents the thermograms of the physical mixture of HA, Bu, and PEG and formulations F5 and F5. A sharp endotherm of pure PEG at 60.5 °C was observed, while in the plot of the physical mixture, it appeared at 59.7 °C. Additionally, a broad exotherm at 206.7 °C was shifted to 203.8 °C [17]. The exotherms of HA were found along the curve of the physical mixture at 221.6 °C and 228.3 °C, which correlate well with previous results [69]. However, the endotherms at 69.0 °C and 124.2 °C were not observed because were overlapped by a strong signal belonging to PEG at 59.7 °C. The set of peaks for Bu were found from 163.1 °C to 192.9 °C, with a sharp endotherm present at 217.6 °C. The maxima from 270.1 °C to 285.4 °C were observed as one peak at 254.0 °C in the thermogram of the physical mixture.

The thermal profile of formulation F5′ shows a broad endotherm at 76.8 °C that may be assigned to HA and PEG, and two exotherms at 226.8 °C and 229.9 °C, attributed to HA. However, the exotherm of PEG at 206.7 °C disappeared, which may be explained by overlapping of the strong signal from HA. The weak PEG exotherm at 202.3 °C appeared again on the curve of the F5 formulation. An interaction between Bu and PEG was not observed. These results are consistent with those of the FTIR study, suggesting that only weak hydrogen bond formation occurred between PEG and HA.

## 4. Conclusions

The results of this study indicate that the addition of a synthetic polymer to an HA-based hydrogel increases its viscosity. The highest viscosity was observed in the case of the formulation doped with AX. This resulted from the interaction between the polymer chains of HA and AX. The release tests confirmed the viscosity results. The dissolution profile of Bu from the formulation that did not contain the synthetic polymer was different from the dissolution curve of Bu released from the hydrogel doped with AX. The drug was released the most slowly from the preparation to which AX was introduced. This makes it a promising hydrogel for intra-articular injection with a prolonged drug release time and, thus, an extended duration of therapeutic activity.

## Figures and Tables

**Table 1:** The compositions of the formulations studied, — mean that this component was not used in the formulation.

Formulation	F1	F1′	F2	F2′	F3	F3′	F4	F4′	F5	F5′
HA % (*w*/*w*)	2	2	2	2	2	2	2	2	2	2
Bu % (*w*/*w*)	1.5	—	1.5	—	1.5	—	1.5	—	1.5	—
Synthetic polymer % (*w*/*w*)	—	—	0.5 PA	0.5 PA	0.5 AX	0.5 AX	0.5 PVA-PVP	0.5 PVA-PVP	0.5 PEG	0.5 PEG

**Table 2:** The dynamic viscosities of the hydrogels at 37 °C and at room temperature (RT) with the pH values.

Formulation	η × 10^−3^ [cP] 37 °C	η × 10^−3^ [cP] RT	pH RT
F1	1.42 ± 0.01	2.11 ± 0.09	6.97 ± 0.04
F1′	2.49 ± 0.04	2.73 ± 0.09	7.18 ± 0.10
F2	3.25 ± 0.07	3.41 ± 0.08	4.36 ± 0.03
F2′	7.30 ± 0.08	7.65 ± 0.07	4.26 ± 0.03
F3	6.45 ± 0.16	6.89 ± 0.05	6.78 ± 0.03
F3′	12.6 ± 0.4	13.1 ± 0.09	6.67 ± 0.01
F4	6.57 ± 0.27	6.84 ± 0.06	6.86 ± 0.07
F4′	3.24 ± 0.01	3.45 ± 0.07	6.82 ± 0.08
F5	6.41 ± 0.35	6.78 ± 0.08	7.05 ± 0.03
F5′	2.99 ± 0.06	3.13 ± 0.05	7.15 ± 0.02

**Table 3:** The difference factor f_1_ and the similarity factor f_2_ obtained by comparing the release profiles of Bu from the F1–F5 hydrogels. The reference formulation was F1. The observed differences between the compared profiles are marked with an asterisk (*).

	f_1_				f_2_			
Formulation	F2	F3	F4	F5	F2	F3	F4	F5
F1	9.1	18.2 *	10.1	3.3	62.6	47.2 *	60.8	82.9

**Table 4:** The kinetic parameters of Bu release from formulations F1–F5.

Kinetic Model	Kinetic Parameters	F1	F2	F3	F4	F5
Z-O	k_0_ × 10^1^ [mg × min^−1^]	3.0 ± 0.3	2.7 ± 0.3	2.4 ± 0.3	2.7 ± 0.3	2.9 ± 0.3
	t_0_._5_ [min]	206.1 ± 19.9	227.3 ± 23.1	253.0 ± 28.6	228.5 ± 22.6	210.8 ± 20.4
	R^2^	0.94 ± 0.02	0.93 ± 0.05	0.91 ± 0.6	0.94 ± 0.01	0.94 ± 0.01
F-O	k_1_ × 10^3^ [min^−1^]	6.4 ± 0.4	4.1 ± 0.3	3.0 ± 0.1	3.6 ± 0.1	4.6 ± 0.9
	t_0_._5_ [min]	133.5 ± 4.2	184.5 ± 9.4	231.4 ± 9.0	192.5 ± 6.7	151.5 ± 2.8
	R^2^	0.98 ± 0.04	0.97 ± 0.04	0.98 ± 0.2	0.99 ± 0.01	0.99 ± 0.01
S-O	k_2_ × 10^4^ [mg^−1^ × min^−1^]	2.6 ± 0.7	1.6 ± 0.5	0.54 ± 0.05	0.80 ± 0.06	1.3 ± 0.2
	t_0_._5_ [min]	53.1 ± 8.9	97.7 ± 9.1	153.6 ± 16.0	105.5 ± 7.4	62.5 ± 7.5
	R^2^	0.81 ± 0.10	0.86 ± 0.19	0.92 ± 0.13	0.96 ± 0.02	0.91 ± 0.02
H	k_H_ [mg × min^−1/2^]	5.3 ± 0.1	4.9 ± 0.2	4.4 ± 0.2	4.8 ± 0.1	5.2 ± 0.1
	t_0_._5_ [min]	134 ± 7.0	160.5 ± 9.6	199.5 ± 17.6	164.4 ± 8.2	139.4 ± 5.5
	R^2^	0.99 ± 0.01	0.99 ± 0.02	0.96 ± 0.05	0.99 ± 0.01	0.99 ± 0.01
K-P	k_K-P_ × 10^2^ [min^–N^]	5.8 ± 0.1	3.9 ± 0.9	3.4 ± 0.2	3.5 ± 0.2	3.2 ± 0.1
	t_0_._5_ [min]	96.0 ± 20.3	128.1 ± 41.6	129.4 ± 11.1	118.7 ± 15.1	123.3 ± 9.1
	R^2^	0.95 ± 0.05	0.85 ± 0.20	0.99 ± 0.01	0.98 ± 0.01	0.99 ± 0.01
	n	0.51 ± 0.02	0.55 ± 0.06	0.54 ± 0.02	0.56 ± 0.03	0.58 ± 0.02
P-S	k_1P-S_ × 10^2^ [min^−m^]	0.2 ± 0.3	1.6 ± 2.4	0.5 ± 0.7	0.02 ± 0.05	0.8 ± 1.4
	k_2P-S_ × 10^2^ [min^−m^]	2.3 ± 0.6	2.3 ± 1.8	2.9 ± 0.6	2.6 ± 1.1	2.5 ± 0.5
	N′	0.31 ± 0.04	0.30 ± 0.07	0.30 ± 0.01	0.33 ± 0.04	0.30 ± 0.05
Best fit		H, K-P	H	K-P	F-O, H, K-P	F-O, H, K-P

k_0_—the zero-order (Z-O) release rate constant; k_1_—the first-order (F-O) release rate constant; k_2_—the second-order (S-O) release rate constant; k_H_—the Higuchi (H) release rate constant; k_K-P_—the Korsmeyer–Peppas (K-P) release rate constant; n—the parameter indicating the drug release mechanism in the Korsmeyer–Peppas equation; t_0_._5_—the half-release time; k_1P-S_—the Peppas–Sahlin release rate constant for the Fickian contribution to the drug release; k_2P-S_—the Peppas–Sahlin release rate constant for the Case II contribution to the drug release; n′—the diffusional exponent in the Peppas–Sahlin equation; R^2^—the correlation coefficient.

## Data Availability

The data supporting the reported results are available from the Department of Physical Chemistry and Biophysics of Wroclaw Medical University.

## Supplementary Materials

The following supporting information can be downloaded at https://www.mdpi.com/article/10.3390/biomedicines13010039/s1: Figure S1: The UV-Vis spectrum of HA; Figure S2: The UV-Vis spectrum of PA; Figure S3: The UV-Vis spectrum of AX; Figure S4: The UV-Vis spectrum of the PVA-PVP mixture; Figure S5: The UV-Vis spectrum of PEG.
