# Supplementary material for: The Effects of Synthetic Polymers on the Release Patterns of Bupivacaine Hydrochloride from Sodium Hyaluronate Hydrogels
Source: Biomedicines. 2024 Dec 27;13(1):39. doi: 10.3390/biomedicines13010039 (PMC11760862; doi:10.3390/biomedicines13010039)
Supplement: Supplementary file 1 [file biomedicines-13-00039-s001.zip › biomedicines-3326419-supplementary.pdf]

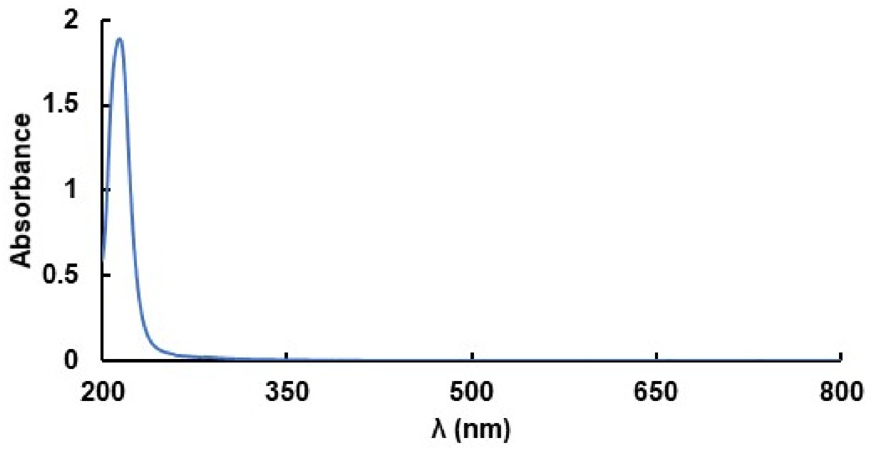

Figure S1. The UV-Vis spectrum of HA.

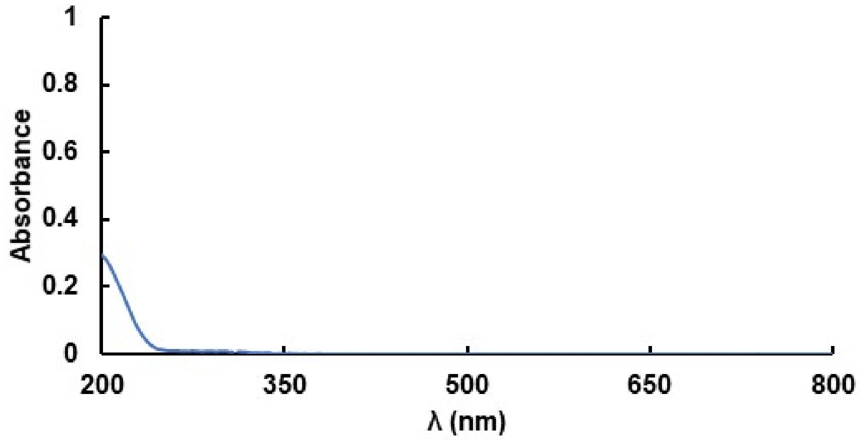

Figure S2. The UV-Vis spectrum of PA.

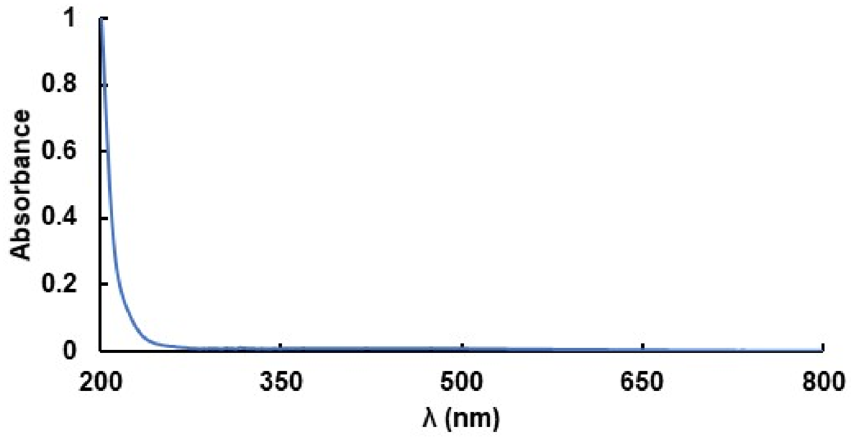

Figure S3. The UV-Vis spectrum of AX.

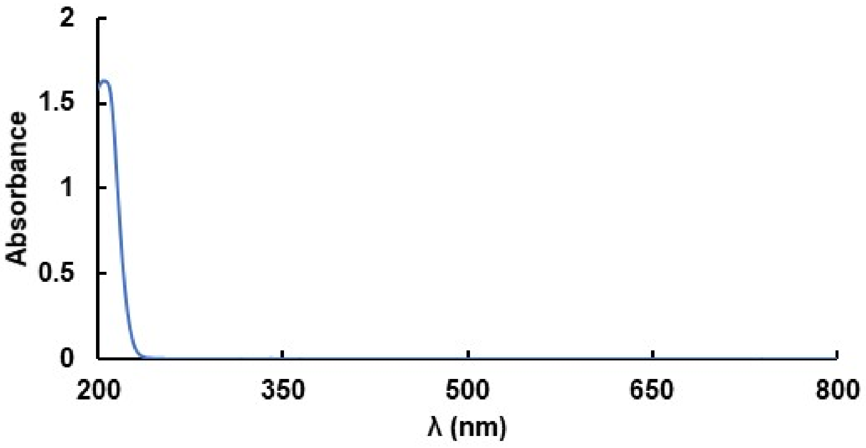

Figure S4. The UV-Vis spectrum of PVA-PVP mixture.

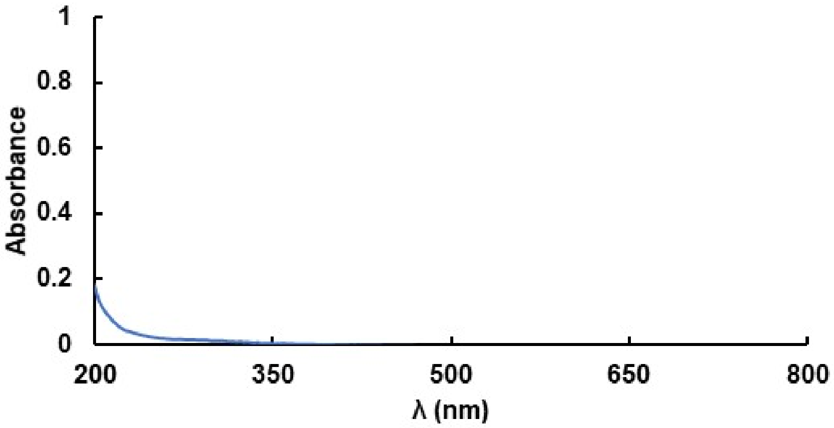

Figure S5. The UV-Vis spectrum of PEG.
